# Temporal Dynamics of Auditory Evoked Neural Oscillations Under a Paired-Pulse Suppression Paradigm

**DOI:** 10.3390/brainsci16020247

**Published:** 2026-02-23

**Authors:** Tomosuke Nakano, Eishi Motomura, Kazuki Hisatomi, Yusuke Nakayama, Kanako Shinke, Takayasu Watanabe, Yasuhiro Kawano, Koji Inui, Motohiro Okada

**Affiliations:** 1 Department of Neuropsychiatry, Mie University Graduate School of Medicine, Tsu 514-8507, Japan; t-nakano@med.mie-u.ac.jp (T.N.); y-kawano@clin.medic.mie-u.ac.jp (Y.K.); okadamot@clin.medic.mie-u.ac.jp (M.O.); 2 Department of Clinical Laboratory, Mie University Hospital, Tsu 514-8507, Japan; clt-kaz-hisatomi@med.mie-u.ac.jp (K.H.); yusuke-n@med.mie-u.ac.jp (Y.N.); k-shinke@med.mie-u.ac.jp (K.S.); takayasu-w@med.mie-u.ac.jp (T.W.); 3 Department of Neuronal Information, Institute for Developmental Research, Aichi Developmental Disability Center, Kasugai 480-0392, Japan; inui@inst-hsc.jp; 4 Section of Brain Function Information, National Institute for Physiological Sciences, Okazaki 444-8553, Japan

**Keywords:** evoked potential, oscillation, paired-pulse suppression, time frequency analysis

## Abstract

**Background/Objectives:** Deficits in auditory change detection are well-known in psychiatric disorders such as schizophrenia. An abrupt change in sound feature during periodic sounds elicits both evoked potentials and a transient change in neural oscillations. Both of these cerebral responses are thought to reflect the automatic change detection. However, the similarities and dissimilarities between these cerebral responses are unclear. To clarify them, we compared the temporal dynamics of evoked potentials and low gamma oscillations under a paired-pulse paradigm. **Methods:** Healthy adults (n = 21) participated. The stimulus was a 2 s sound consisting of a train of 25 ms pure tones. The sound pressure was increased by 15 dB twice within a 600 ms interval. Electroencephalographic signals were recorded from Fz and Cz electrodes referenced to linked mastoids. The peak (N100)-to-peak (P200) amplitude and the inter-trial phase coherence (ITPC) of low gamma oscillations were analyzed. **Results:** Auditory steady-state responses were evoked around 40 Hz. An abrupt change in sound pressure transiently decreased the ITPC of the oscillations at 40 Hz, whereas it increased the ITPC at the remaining frequencies. Unlike the change-related potentials, the degree of ITPC responses did not differ between the two changes. **Conclusions:** The synchrony of low gamma oscillations transiently responded to an abrupt increase in sound pressure but did not show paired-pulse suppression. This novel neurophysiological approach enables a focus on the neural change detection from multiple angles, which could be useful for investigations of psychiatric disorders.

## 1. Introduction

Electroencephalography (EEG) and magnetoencephalography (MEG) with high temporal resolution are useful tools for investigations of auditory processing impairments in individuals with psychiatric disorders. Auditory stimuli at specific frequencies increase the amplitude of neural oscillations at the stimulus frequency, with 40 Hz stimuli producing the greatest effect [1]. Ross et al. revealed that when a 40 Hz stimulus is presented, the 40 Hz oscillation amplitude gradually increases for up to 250 ms after the onset of the sound [2]. The 40 Hz oscillation then reaches a steady state that is called the ‘auditory steady-state response (ASSR)’. After Kwon et al. first described a reduction in the 40 Hz ASSR in patients with schizophrenia [3], many studies further examined this reduction in patients with schizophrenia and other psychiatric disorders. Meta-analysis studies have revealed significant reductions in the amplitude and inter-trial phase coherence (ITPC) of the 40 Hz ASSR in patients with schizophrenia [4] and bipolar disorder [5]. The deficits of neural synchrony under 40 Hz sound stimulation seem to indicate one of the common backgrounds among these psychiatric disorders.

An abrupt change in the middle of a periodic sound, such as a stimulus omission/gap [6,7], an inter-phase difference [8,9], or a short noise burst [10], causes a transient change in the phase of the 40 Hz ASSR. In light of the finding that the degree of the 40 Hz event-related desynchronization (ERD) depended on the magnitude of the changes in sound pressure regardless of whether the sound pressure increased or decreased [11], we speculate that the 40 Hz ERD reflects the automatic change detection.

Deficits of pre-attentive auditory processing, such as change detection and sensory gating in psychiatric disorders, is generally accepted. In regard to neural change detection, auditory evoked potentials and magnetic fields are generally used to investigate auditory processing in individuals with psychiatric disorders. Cerebral responses to a deviant stimulus embedded within a sequence of repetitive standard stimuli have been observed; these cerebral responses are called mismatch negativity (MMN), which is a well-established evoked component. Numerous studies have reported a reduced MMN amplitude in psychiatric disorders [12,13,14]. In addition, various types of abrupt changes in the sound feature during an ongoing sound elicited triphasic evoked potentials at approx. 50–250 ms after the change onset such as sound pressure [15,16], sound location [15,17,18], sound frequency [15,19,20,21] and gap [22]. Similar to MMN, this cerebral response depends on the magnitude of the change and is based on the echoic memory. This response, consisting of three components (P50, N100 and P200), is called the ‘change-related response’ [15]. In regard to sensory gating, a paired-pulse suppression (PPS) paradigm has been applied in psychiatric research. When two identical sound stimuli are presented at fixed intervals, the late responses (i.e., the P50, N100, and P200) elicited by the second stimulus are smaller than those elicited by the first stimulus. The measurement of the difference between the responses to the first and second stimuli is thought to reflect neural inhibitory processes that prevent an overflow of sensory inputs. Impaired sensory gating has been reported in schizophrenia [23,24,25,26], bipolar disorder [25,26] and autism spectrum disorder [27].

Our research group established a PPS paradigm that uses a train of 25 ms long amplitude-moderate tones with an abrupt increase in sound pressure that occurs twice [28]. Unlike PPS paradigms with a silent interval, this PPS paradigm at a 40 Hz driving frequency enables the evaluations of neural oscillation, as well as evoked potentials. We considered that this stimulus paradigm could be a useful tool for clinical research to investigate auditory processing with multiple angles. However, the effect of abrupt changes in the sound feature on neural oscillations has not been thoroughly investigated. Before clinical research can be conducted, two questions need answers: (1) Whether or how do the gamma frequencies other than 40 Hz behave in response to sudden changes in the sound feature? (2) Whether the filtering of the information inputs would be observed in neural oscillation similar to that observed for evoked potentials? We used a PPS paradigm in the present study to address the two questions presented above, which are important for understanding the neural basis of sensory processing impairments in psychiatric disorders.

## 2. Materials and Methods

### 2.1. Subjects

Twenty-one healthy volunteers (13 males and eight females; mean age 30.0  ±  9.5 years) with normal hearing participated in the study. All were right-handed, as determined by the Edinburgh Handedness Inventory [29]. None of the subjects had any history of neurological or psychiatric disorders or substance abuse. The study was approved by the Clinical Research Ethics Review Committee of Mie University Hospital (approval no. H2022-198, 26 October 2022). Written consent to participate was obtained from each subject before the experiment.

### 2.2. Stimuli

**Figure 1:**
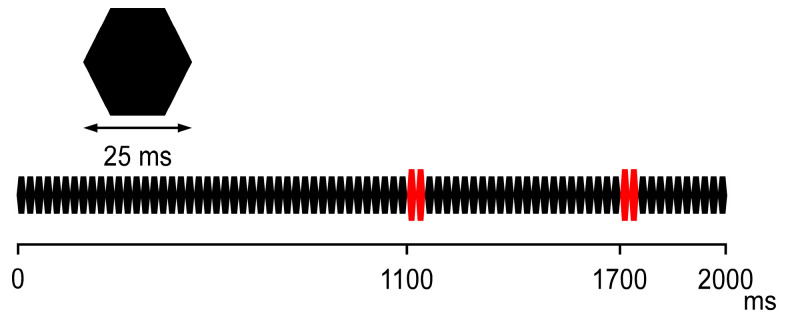
The sound stimuli used in this study. The two consecutive tones (red), occurring 1100 and 1700 ms after the sound onset, increase the sound pressure by 15 dB from a baseline of 65 dB.

A 2 s sound consisting of a train of 25 ms long amplitude-modulated pure tones (800 Hz; rise/fall: 5 ms) with a pair of changes in the sound pressure (Figure 1) was used as described elsewhere [28]. The sound stimuli were delivered to the subject via a headphone with an inter-stimulus interval of 500 ms. Before recording, the subjects confirmed that they detected the change in sound pressure (instead of audiometry testing). During the experiment, the subjects watched a silent movie to ignore the presented sound.

### 2.3. EEG Recording

In a quiet electrically shielded room, EEG signals were recorded at a sampling rate of 1000 Hz with an analog filter of 0.1–120 Hz. The scalp electrodes were placed at Fp1/Fp2, Fpz, F3/F4, Fz, C3/C4, Cz, P3/P4, Pz, O1/O2, F7/F8, T3/T4, T5/T6, and P9/P10 according to the international 10/10 system. Two additional electrodes were placed above the right lateral canthus and below the left lateral canthus so that trials with eye blinks could be omitted from the subsequent analyses. The impedance of electrodes was kept bellow 5 kΩ. Epochs with a voltage value larger than 100 µV in any electrode were rejected.

At least 100 artifact-free trials with a time window from 200 ms before to 2300 ms after the sound onset were averaged in each subject, with an offline filter of 0.16–100 Hz. The Fz and Cz electrodes referring to the linked mastoids (P9 and P10) were used as exploring electrodes.

### 2.4. Data Analysis Procedure

The brain electric source analysis software package (BESA 6.0, Grafefling, Germany) was used to analyze the subjects’ EEG signals. A 0.16 to 35 Hz band-pass filter was used for the analysis of evoked potentials. The pre-stimulus period (from 200 to 100 ms before the sound onset) was used as the DC offset. The targeting evoked potential was the Change-N100 peaking at 65–160 ms and a following positivity (P200) peaking at 150–270 ms. To avoid the effects of baseline shift, the peak-to-peak amplitude of N100 and P200 was used as the Change-N100 amplitude [15]. The degree of PPS was defined as (the amplitude of the first response − the amplitude of the second response)/the amplitude of the first response × 100.

**Figure 2:**
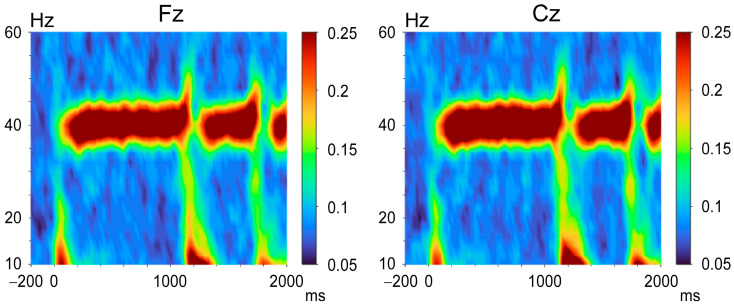
The grand averaged time-frequency maps of the inter-trial phase coherence (ITPC). Color bars indicate the ITPC at each time-frequency point.

We conducted time frequency analyses (TFAs) at frequencies ranging from 4 to 100 Hz in 2 Hz steps using a Morlet wavelet approach with five cycles. After the Morlet wavelet transform was applied to the recorded EEG data at 25 ms intervals, the ITPC was calculated. The ITPC values ranged from 0 (no phase synchronization) to 1 (complete phase synchronization). The averaged ITPC from 200 to 100 ms before the sound onset was used as the pre-stimulus value. The grand-averaged time-frequency map across all subjects is shown in Figure 2 for illustrative purposes.

The strength of 40 Hz ASSR varied until 250 ms after sound onset, after which they reached a steady state. The 40 Hz ASSR consists of an initial transient response and a late steady-state response from the perspective of the ITPC’s temporal dynamics [2,10]. Several studies selected and investigated the later part of the steady state [30,31,32,33]. To avoid contamination from early transient responses, we averaged the ITPC values during the late steady-state period between 350 and 850 and compared with these values with the pre-stimulus baseline values to determine whether the steady-state response was significant.

For the evaluation of the effect of the abrupt sound pressure change on neural oscillations, we selected the minimum or maximum value of ITPC following the first and second pulses during the periods of 1100–1350 ms and 1700–1950 ms after the sound onset, respectively. The difference between the minimum and maximum values from the averaged ITPC during the pre-pulse oscillation (i.e., 850–1000 and 1450–1600 ms after the sound onset) was then calculated.

### 2.5. Statistical Analyses

According to our previous studies, the effect size was 0.86 for PPS of the change-related potentials [28] and 1.52 for ITPC of 40 Hz ASSR [34]. The sample size of the present study was determined by a priori analysis using Wilcoxon signed rank test design (G*Power 3.1; n = 21 with two tails, normal distribution, effect size = 0.86, α error probability = 0.05, power = 0.95). Since the Shapiro–Wilk test did not show a normal distribution of ITPC data at almost all of the frequency bands, the non-parametric Wilcoxon signed-rank test was used in this study. The significance of the oscillation entrained to the stimulation rate was confirmed by comparisons of the ITPC values between the pre-stimulus baseline and the ASSRs. To evaluate the significance of the change in ITPC, we compared the minimum/maximum values with those of a pre-pulse period. To evaluate the effects of paired pulses, we compared the amplitude of Change-N100, as well as the degree of ITPC changes between the two pulses. The data are presented as the mean ± standard deviation. Probability (*p*)-values ≤ 0.05 were considered significant. The statistical analyses were carried out using BellCurve for Excel version 4.09 (Social Survey Research Information Co., Tokyo, Japan).

## 3. Results

**Figure 3:**
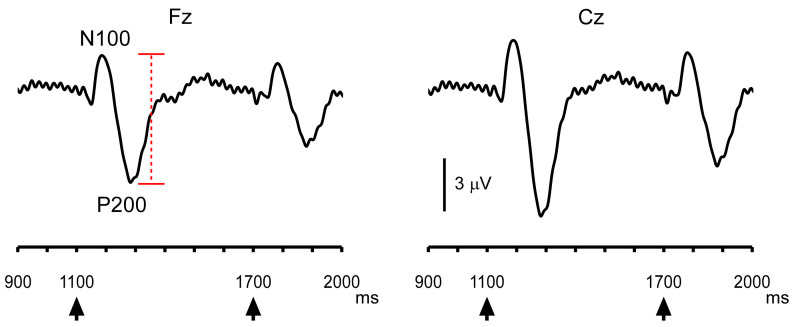
The grand-averaged evoked potential waveforms. Arrowheads indicate the timing of the increase in sound pressure. A red dashed line indicates the peak-to-peak amplitude.

**Figure 4:**
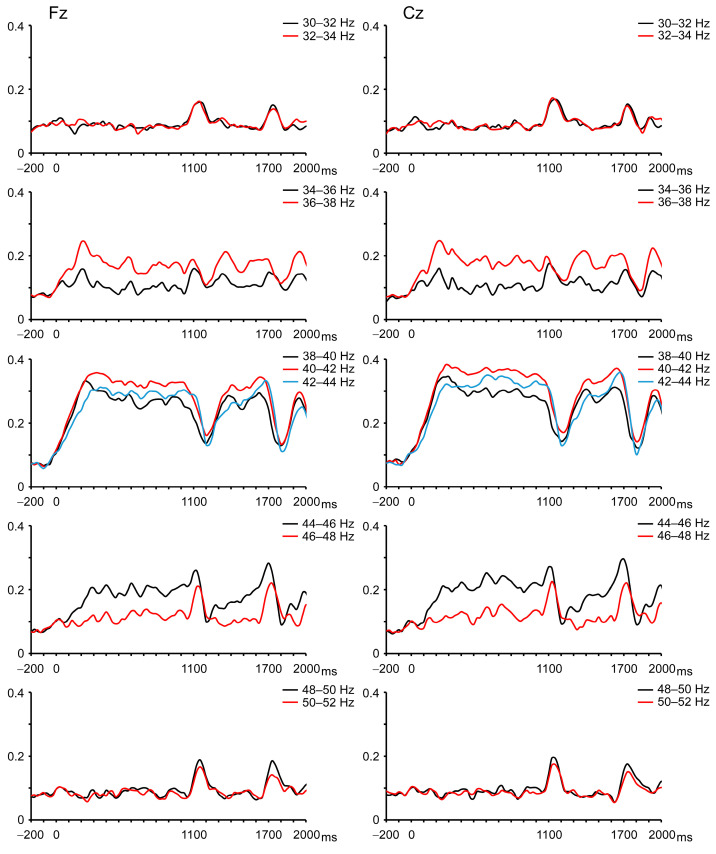
The grand-averaged time courses of the ITPC of the neural oscillations in each frequency’s low gamma band.

The abrupt increase in sound pressure elicited change-related auditory potentials, with the second response being smaller than the first response (Figure 3). For instructive purposes, the grand-averaged ITPC time courses of oscillations at each frequency are shown in Figure 4. Increases in ITPC at the stimulus frequency were readily observed.

The data for each frequency band are listed in Table 1. Compared to the pre-stimulus baseline, a significant increase in ITPC was observed for 34 to 46 Hz oscillations at Fz and for 34 to 48 Hz oscillations at Cz. Similar to evoked potentials, the change in sound pressure caused changes in neural oscillations, as demonstrated by the time courses of the ITPC for each frequency band. However, the effect on oscillations was not straightforward. At around 40 Hz (38–44 Hz), the ITPC was significantly decreased by the change in sound pressure, and it then returned to the steady-state level (Figure 4). That is, the change in sound pressure decreased the ITPC of the 40 Hz ASSR. In contrast, the ITPC was significantly increased at oscillations at 30–34 and 48–52 Hz. At the remaining frequencies, i.e., 44–48 and 34–38 Hz, the ITPC exhibited a biphasic response—a slight increase and then a decrease, that is, a combination of the two aforementioned responses.

**Figure 5:**
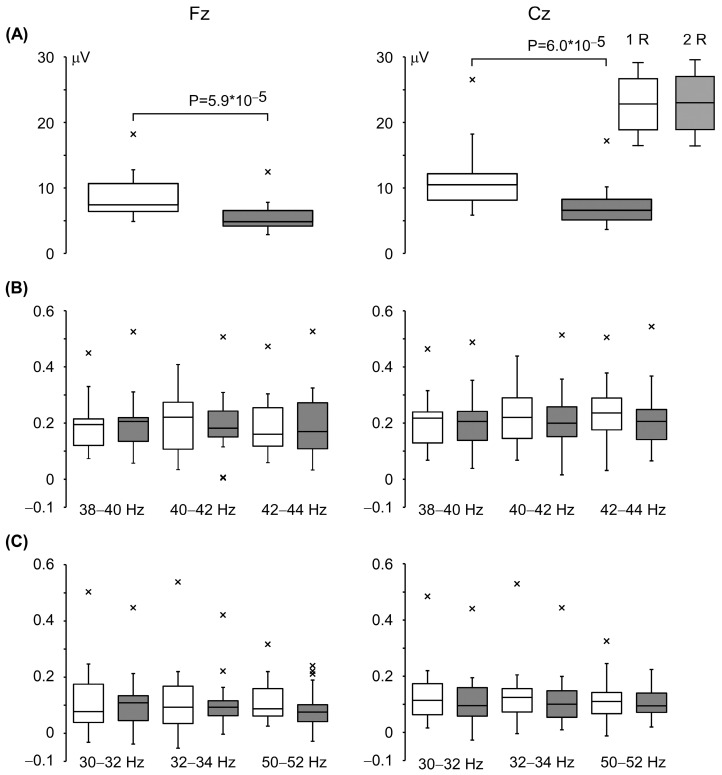
Comparison of responses to the first and second changes in sound pressure. The top and bottom of each box: the 75th and 25th percentiles of the Change-N100 amplitude (**A**) and the degree of decrease (**B**) and increase (**C**) in ITPC from the pre-pulse level. The horizontal lines in the boxes indicate the median. The whiskers extend 1.5 times the interquartile range from the 25th and 75th percentiles. The labels ‘1R’ and ‘2R’ refer to the responses to the first and second changes in sound pressure, respectively. Points beyond the whiskers: outliers.

The box plots in Figure 5 depict the Change-N100 amplitudes and the ITPC differences between the pre-pulse level and the maximum/minimum values at the representative frequencies. The degree of PPS in the Change-N100 amplitudes averaged 34.6  ±  11.2% at Fz and 34.7  ±  10.6% at Cz. Our comparison of the changes in oscillation between the two pulses revealed no significant difference at any frequency band, unlike the result that has been observed for evoked potentials.

## 4. Discussion

The majority of 40 Hz ASSR studies have focused on ASSR values at the stimulation frequency (40 Hz). The behavior of the neural oscillatory response to an abrupt increase in sound pressure at surrounding frequencies seems interesting (Figure 2). Considering this, we focused on the time course of ITPC at low gamma frequencies (30–52 Hz) in detail. This study revealed two major findings: (i) an abrupt increase in sound pressure caused a transient change in low gamma oscillations, and (ii) in line with our MEG study using the same stimulus interval (600 ms) [35], PPS was clearly observed in the change-related potentials, but not in the transient ITPC changes (Figure 4).

We observed that the ITPC of the 40 Hz ASSR transiently decreased after the change onset, as reported in a MEG study [11]. As for evoked potentials, the onset, offset, and abrupt changes in sound feature elicit change-related responses, based on the comparison between the prior and novel states. The neural origin of these cerebral responses was estimated to be in the superior temporal gyrus, with no differences observed among the three events (i.e., onset, offset and abrupt changes in sound feature). In addition, the amplitude of the responses to these three events showed a similar manner against the duration of the preceding state [36]. Similarly, it is known that an abrupt sensory change affects the 40 Hz ASSR, producing transient decreases in the amplitude and the ITPC [11]. Of note, these responses of oscillations occurred when a control stimulus with no changes was rarely presented among frequent stimuli with sound feature’s change [11]. It is possible that the oscillations were modulated not only by neural inputs from the peripheral nerves, but also by higher-order neural circuits, as has been suggested for evoked potentials [15,36].

Interestingly, we observed that the ITPC of low gamma oscillations (other than at 40 Hz) transiently increased after the change in sound pressure. Similar to the present results, a recent MEG study revealed that the ITPC increased significantly in broad-band frequencies after the onset and offset of a 40 Hz sound [37]. In other words, a sudden change in sound feature, including an offset, could temporarily increase the synchrony in low-to mid-gamma activity. A question then arises as to why the ITPC decreased at 40 Hz and exhibited a biphasic response moving away from 40 Hz in the present study. It has been established that gamma oscillations are involved in various types of cognitive function [38], and thus one possible mechanism is that the oscillation network for auditory-change detection differs from that entraining to the driving frequency. It therefore seems reasonable that the network for auditory-change detection was prioritized for survival when abrupt changes in sound features occurred. These two networks are similar in oscillation frequency but may not be similar in response to a sensory change event. This resulted in solely increasing/decreasing or biphasic ITPC responses.

Concerning the PPS results observed herein, one possible reason for the discrepancy in the filtering of the information inputs between change-related responses and neural oscillations is the difference in the responsible cortical area between the change-related response and the ASSR. The dipole origin of auditory Change-N100m (the main component of change-related response) has been estimated to be at the superior temporal gyrus (secondary auditory cortex: A2) [36]. Several MEG studies revealed that the main ASSR generator appeared to be located at the primary auditory cortex (A1), which is medial to the N100m source [2,39,40]. In support of this notion, Matsubara et al. recorded ASSRs from electrodes implanted at Heschel’s gyrus in patients with temporal epilepsy [41]. The P30 origin, which occurs earlier than P50, was located in the primary auditory cortex [42]. Sensory gating was observed in the P50m component, but not in the P30m component [43]. Similarly, the origins of the somatosensory-evoked magnetic fields were estimated at the postcentral gyrus (S1) in the contralateral hemisphere and the perisylvian region (S2) in both hemispheres, and then, PPS is clearly observed in S2 but not in S1 [35,44]. Considering the serial and hierarchical sensory processing, it seems reasonable that PPS occurs in higher-order processing areas but not in primary sensory areas. We speculate that this explanation could apply to the present results of auditory system, which showed a difference in PPS between evoked potentials and oscillations.

Methodological considerations should be discussed. First, our paradigm employs the repetition of 25 ms tones with an abrupt increase in sound pressure that occurs twice. In the traditional paired-clicks paradigm with a silent interval, the time interval between two pulses affects the degree of PPS [45]. Considering this, one of our earlier studies confirmed that the interval of 600 ms most effectively suppresses the change-related response [46]. However, the present study was not parametric and it needs to be confirmed whether this stimulus interval is favorable for observing PPS effects on neural oscillations. It is also unclear whether the 15 dB increase in sound pressure was sufficient. Second, a repetition of 25 ms tones (i.e., 40 Hz auditory stimulation) makes it possible to observe steady-state responses during pre-pulse periods at the driving frequency and transient changes evoked by an abrupt change in sound pressure at gamma frequencies as well. Lastly, the recording time used in the present sound paradigm is much shorter (in 6–7 min) compared to that in the pair-clicks paradigm commonly used in PPS studies. The duration of the stimulus used in the present study was relatively long (2 s) itself, but the trial-to-trial interval was only 500 ms, which is far shorter than that of the pair-clicks paradigm (8–12 s) used in PPS studies. In the pair-clicks paradigm, the echoic memory established by the first click pulse gradually decreases in silence; it takes a long time to return to baseline levels and results in the relative long recording time. The short-term recording, an advantage of the present paradigm, is quite important for patients with psychiatric disorders.

There are several study limitations to address. First, as has been done in the majority of ASSR studies, we used only 40 Hz stimulation, which is considered the best resonant frequency stimulus for auditory neural circuits. Other stimulation rates, e.g., 30 Hz, could also entrain neural oscillations, and it was reported that individuals with schizophrenia showed reductions in the 30 Hz ASSR [4]. It is thus of interest to determine whether the change-evoked transient alteration in oscillations is specific to 40 Hz stimulation or can be observed under other stimulation rates. Second, the data of this study were obtained from just two electrodes (Fz and Cz) because ASSRs and change-related responses recorded by EEG are front-central prominent potentials due to the generator located at auditory cortex, as clarified by MEG studies [36,47]. Third, this study did not sufficiently control for attentional state. A functional magnetic resonance imaging study revealed that attention modulates neural activity in the primary and secondary cortex [48]. Several studies have revealed that the 40 Hz ASSR is influenced by attention to the ASSR [49,50]. Interestingly, a study revealed that patients with schizophrenia lacked the ability to modulate their 40 Hz ASSR through attention [51]. However, a review has reported that there is no consensus regarding the effect of attention on the ASSR [52]. It should also be confirmed that the transient change in neural oscillations caused by an abrupt change in sound pressure has an attention effect. Finally, the sample size was relatively small (21 subjects). While the generation of 40 Hz ASSR or PPS of the evoked potentials is robust and the current sample size fell within a safe range, a larger sample might have been necessary to detect paired stimulation effects on neural oscillations.

It would be desirable to conduct further studies using EEG signals obtained from multiple electrodes and employing emerging analytical approaches, such as brain network analysis and cross-frequency coupling. An EEG study suggested the possibility of an association between dysfunction of the brain connectivity of the ASSR and hallucination in schizophrenia [53]. In addition, applying deep learning to the present results is recommended [54]. Each of the above-mentioned issues should be investigated in future studies.

## 5. Conclusions

By using a 40 Hz sound with an abrupt increase in sound pressure provided twice at 600 ms intervals, we investigated the temporal dynamics of low gamma oscillations, as well as change-related potentials. We observed that the ITPC of 40 Hz oscillations decreased transiently around 40 Hz, whereas it increased at the remaining frequencies. Unlike change-related potentials, the degree of ITPC responses to the second change stimulus was the same as the degree of the responses to the first change stimulus; that is, ITPC responses did not show PPS. The present paradigm enables us to analyze auditory change detection from multiple angles, providing a new perspective on cognitive impairment in psychiatric disorders.

## Figures and Tables

**Table 1:** Inter-trial phase coherence (ITPC) values.

Frequency, Hz	Mean, 850–1000 ms	Max. After 1st Pulse	*p*-Value	Min. After 1st Pulse	*p*-Value	Mean, 1450–1600 ms	Max. After 2nd Pulse	*p*-Value	Min. After 2nd Pulse	*p*-Value
Fz										
30–32	0.08 (0.02)	0.20 (0.11)	1.2 × 10^−4^	―	―	0.08 (0.03)	0.19 (0.10)	1.6 × 10^−4^	―	―
32–34	0.08 (0.03)	0.19 (0.11)	1.4 × 10^−4^	―	―	0.08 (0.03)	0.19 (0.18)	6.9 × 10^−5^	―	―
34–36	0.11 (0.02)	0.18 (0.12)	0.003	0.05 (0.03)	6.9 × 10^−5^	0.11 (0.04)	0.18 (0.09)	3.2 × 10^−4^	0.05 (0.03)	6.0 × 10^−5^
36–38	0.17 (0.07)	0.19 (0.14)	0.90	0.06 (0.04)	6.9 × 10^−5^	0.16 (0.08)	0.20 (0.13)	0.19	0.06 (0.03)	6.0 × 10^−5^
38–40	0.27 (0.11)	―	―	0.08 (0.05)	6.0 × 10^−5^	0.26 (0.12)	―	―	0.07 (0.05)	6.0 × 10^−5^
40–42	0.32 (0.12)	―	―	0.13 (0.08)	6.0 × 10^−5^	0.31 (0.13)	―	―	0.11 (0.07)	6.0 × 10^−5^
42–44	0.29 (0.12)	―	―	0.10 (0.05)	6.0 × 10^−5^	0.27 (0.13)	―	―	0.08 (0.06)	6.0 × 10^−5^
44–46	0.19 (0.09)	0.27 (0.11)	3.7 × 10^−4^	0.06 (0.05)	6.0 × 10^−5^	0.18 (0.10)	0.28 (0.12)	2.1 × 10^−4^	0.06 (0.03)	6.0 × 10^−5^
46–48	0.12 (0.03)	0.22 (0.09)	1.2 × 10^−4^	0.05 (0.03)	1.1 × 10^−4^	0.10 (0.03)	0.23 (0.09)	8.0 × 10^−5^	0.06 (0.04)	4.2 × 10^−4^
48–50	0.09 (0.03)	0.21 (0.09)	6.9 × 10^−5^	―	―	0.08 (0.03)	0.21 (0.08)	6.9 × 10^−5^	―	―
50–52	0.08 (0.02)	0.19 (0.08)	6.9 × 10^−5^	―	―	0.09 (0.03)	0.18 (0.07)	8.0 × 10^−5^	―	―
Cz										
30–32	0.08 (0.02)	0.21 (0.11)	6.0 × 10^−5^	―	―	0.08 (0.03)	0.19 (0.09)	1.0 × 10^−4^	―	―
32–34	0.08 (0.02)	0.21 (0.10)	6.9 × 10^−5^	―	―	0.08 (0.02)	0.19 (0.09)	6.0 × 10^−5^	―	―
34–36	0.10 (0.02)	0.21 (0.10)	1.1 × 10^−4^	0.04 (0.03)	8.0 × 10^−5^	0.09 (0.03)	0.18 (0.09)	6.0 × 10^−5^	0.05 (0.03)	1.1 × 10^−4^
36–38	0.18 (0.05)	0.21 (0.12)	0.48	0.07 (0.04)	6.9 × 10^−5^	0.17 (0.07)	0.21 (0.11)	0.01	0.05 (0.04)	6.9 × 10^−5^
38–40	0.29 (0.10)	―	―	0.08 (0.06)	6.0 × 10^−5^	0.28 (0.11)	―	―	0.07 (0.04)	6.0 × 10^−5^
40–42	0.35 (0.12)	―	―	0.13 (0.07)	6.0 × 10^−5^	0.33 (0.13)	―	―	0.12 (0.07)	6.0 × 10^−5^
42–44	0.32 (0.11)	―	―	0.08 (0.05)	6.0 × 10^−5^	0.29 (0.11)	―	―	0.08 (0.05)	6.0 × 10^−5^
44–46	0.21 (0.08)	0.29 (0.10)	0.001	0.06 (0.03)	6.0 × 10^−5^	0.18 (0.08)	0.31 (0.11)	1.6 × 10^−4^	0.06 (0.03)	6.0 × 10^−5^
46–48	0.12 (0.03)	0.24 (0.10)	1.4 × 10^−4^	0.05 (0.04)	9.2 × 10^−5^	0.11 (0.04)	0.25 (0.08)	6.9 × 10^−5^	0.06 (0.03)	6.9 × 10^−5^
48–50	0.09 (0.03)	0.22 (0.08)	6.0 × 10^−5^	―	―	0.08 (0.04)	0.21 (0.07)	6.0 × 10^−5^	―	―
50–52	0.08 (0.03)	0.20 (0.07)	6.9 × 10^−5^	―	―	0.08 (0.04)	0.19 (0.07)	6.0 × 10^−5^	―	―

Data are mean (standard deviation).

## Data Availability

The data presented in this study are available on request from the corresponding author due to privacy reasons.

## Abbreviations

The following abbreviations are used in this manuscript:

EEG	Electroencephalography
MEG	Magnetoencephalography
ASSR	Auditory steady-state response
ITPC	Inter-trial phase coherence
ERD	Event-related desynchronization
MMN	Mismatch negativity
PPS	Paired-pulse suppression
